# A scoring system as a method to evaluate pilonidal sinus disease to make an easy decision for its management

**DOI:** 10.4103/0970-0358.53011

**Published:** 2009

**Authors:** Mohamed M. S. Awad, Amr Abd Elbaset, Samir Ebraheem, Esmael Tantawy, M. Abd Elhafez, Atia M. Elsayed

**Affiliations:** Department of Surgery, Zagazig University Hospitals, Egypt

**Keywords:** Pilonidal sinus, Recurrent sinuses, Scoring system

## Abstract

This study was planned to evaluate prospectively the results of 150 patients with pilonidal sinus treated surgically after planning a score system. The aim was to choose the proper technique for the proper pilonidal sinus disease. From November 2002 to December 2006, 150 patients [130 male, 20 female; average age = 22.65 ± 4.2 (range, 15-46) years] with primary or recurrent pilonidal sinus diseases, operated under spinal anaesthesia or general anaesthesia. All patients were classified into three groups A, B and C. According to the clinical presentations we had three groups of patients: Group A with a score number n = 8 to 10, Group B with a score number n = 11 to 13, and Group C had the number n = 14 to 16. The study helped us in our surgical decision making, particularly in choosing the type of surgery for a given patient by our new scoring system, with promising results and low failure rate.

## INTRODUCTION

The surgical management of sacrococcygeal pilonidal sinus is still a matter of discussion. Therapy ranges from complete wide excision with or without closure of the wound to just an excision of the sinus. Also the aetiology of the disease, either congenital or acquired still needs more discussions. Initially, pilonidal cysts were believed to be congenital in nature. This theory has been called into question for multiple reasons.[1] Multiple case reports described pilonidal cyst formation in jeep drivers in World War II. So many servicemen were affected with pilonidal disease that it was renamed “jeep disease.” These findings led to the belief that pilonidal cysts can be acquired by excessive repetitive trauma to the sacrococcygeal region.[2] Though pilonidal sinus disease has been surgically treated for more than 100 years, its management remains controversial and many reports[3,4] have advocated various different approaches.

The aim of this study is to create a scoring system for simple dealing with pilonidal sinus diseases regarding its clinical presentations.

## MATERIAL AND METHODS

**Figure 1:**
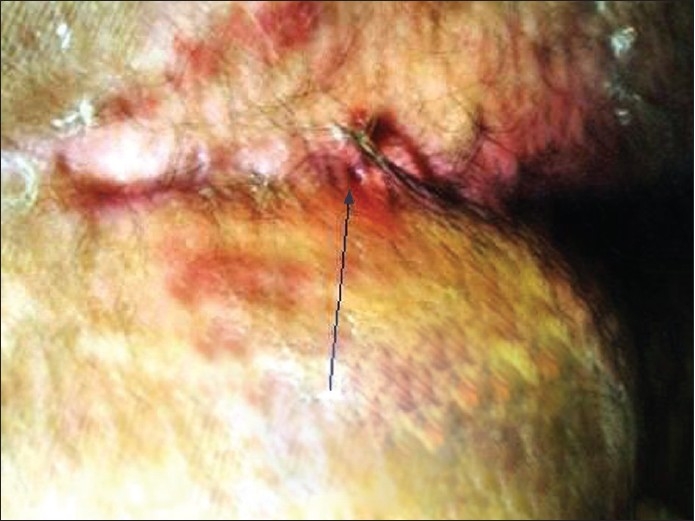
Pilonidal dimple attracts hair within itself

**Figure 2:**
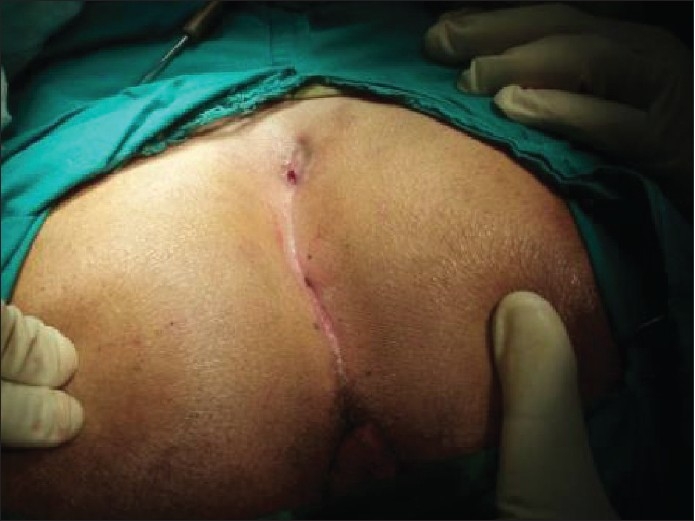
Pilonidal dimple and midline sinuses

**Figure 3:**
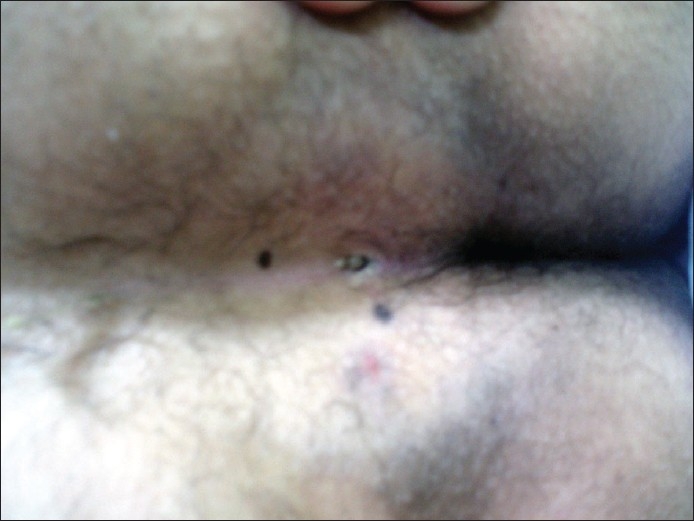
Pilonidal dimple without sinus containing necrotic debris

**Figure 4:**
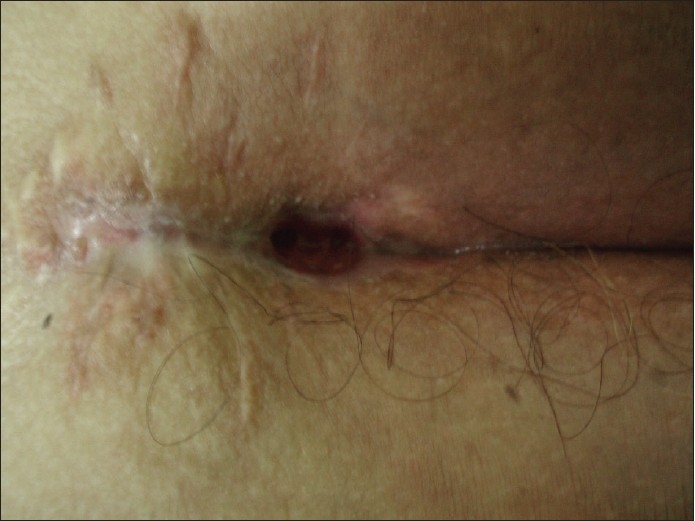
Late recurrence after one year

**Figure 5:**
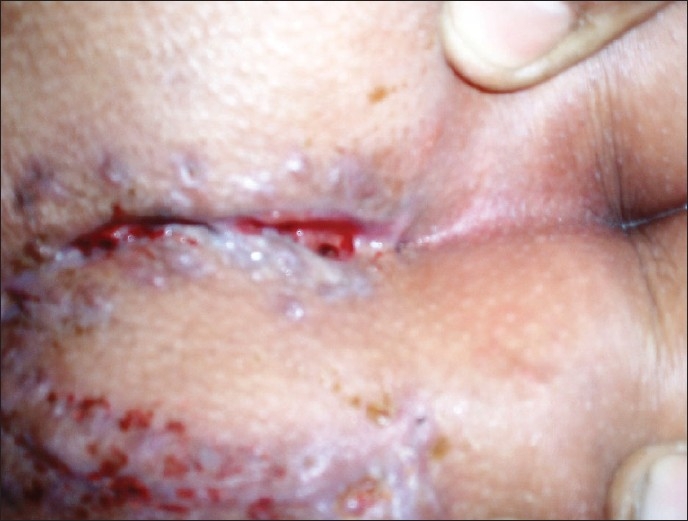
Early recurrent sinus due to persistence of the dimple

**Figure 6:**
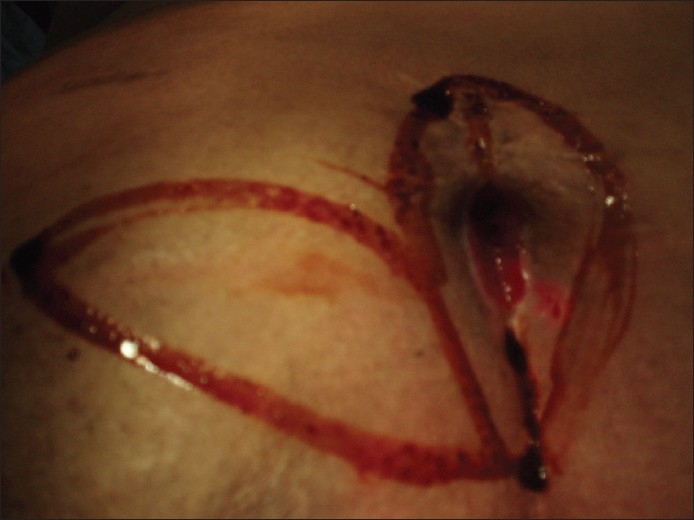
Marking the rotation flap and the eccentric excision

**Figure 7:**
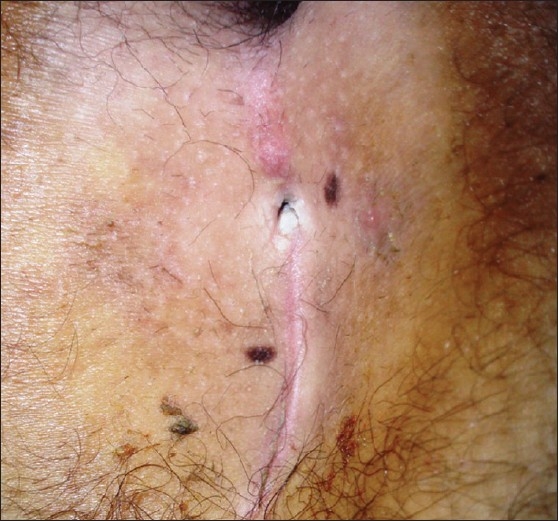
Male patient with dimple attracts hair and necrotic debris

**Figure 8:**
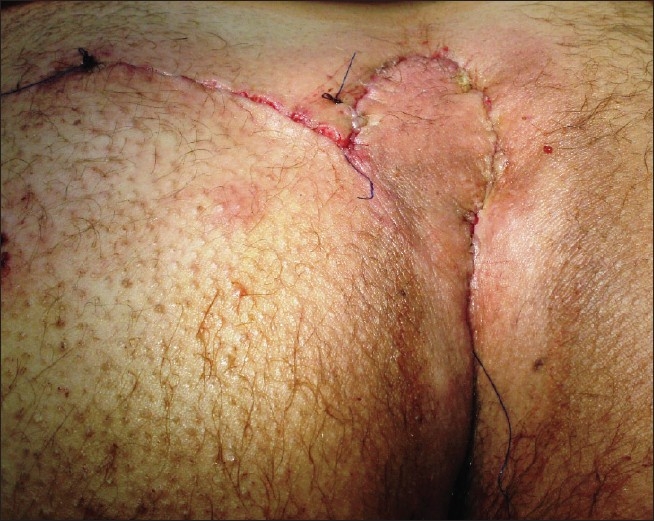
After closure with rotation flap

One hundred and fifty patients were admitted in the surgical department of Zagazig University hospitals, with pilonidal sinus diseases in the period between November 2002 to December, 2006. The preoperative patient characteristics were collected and summarized in a certain fashion like the sex, weight, hirsute, also the sinuses' characteristics were documented including, the number, site, size, duration of the sinus disease and either primary or recurrent [Table 1]. We gave each characteristic, a score number, either one or two according to the severity of the disease [Figures 1–8].[1–6]

**Table 1:** Preoperative patient characteristics and scoring numbers

*The characteristics*	*Score number[2]*	*Score number[1]*
Patients' characteristics		
Hirsute	Hairy (coarse with hard texture)[2]	Less (Faint hair with fine texture)[1]
Weight	Overweight[2]	Average weight or under[1]
Sex	Male[2]	Female[1]
Sinus characteristics		
Number of the sinuses	Multiple![2]	Single[1]
Site	At the convex side[2]	At the midline[1]
Size	<0.5[2]	>0.5[1]
Recurrence®	Recurrent[2]	Primary[1]
Duration	More than 6 months[2]	Less than 6 months[1]
Total score	[16]	[8]

Patients with a score number from 8 to 10 [Score A]: n = 47 – They were managed with local excision and conservative treatment (Excision and healing by secondary intention): Limited excision to remove the inflamed tissue and debris allowing the wound to granulate from its base. This procedure necessitated local anaesthesia.

Patients with a score number from 11 to 13 [Score B]: n = 53 – They were suitable for local excision and primary closure. An elliptical eccentric excision including all midline pits and a release of the underlying tissues on both sides above presacral fascia was followed primary repair in two layers. This procedure necessitated spinal or general anaesthesia.

Patients with a score number from 14 to 16 [Score C]: n = 50 – After excision, the defent could not be closed primarily but required flap coverage. We had selected unilateral or bilateral rotation flaps for this purpose[Figures 6–8].

All the patients of Group B and C were placed on the left lateral position or prone position after administering regional or general anaesthesia. Parentral preoperative broad-spectrum antibiotics Cefobid (cefoperazone) and Metronidazole were given and repeated during the procedure. Post-operatively alternate sutures were removed on the tenth day and once there was no wound discharge, the remaining sutures were removed after two weeks.

## RESULTS

### Excision and healing by secondary intention [n = 47]

All the patients had no hospital stay or long bed rest; however, the downside was a lengthy healing time (three to eight weeks) with an average of five weeks – the principal advantage being a low recurrence rate [2.1%] [Table 2].

**Table 2:** Postoperative patients' characteristics after follow-up

*Postoperative patients' characteristics*	*Patients with score A [n = 47]*	*Patients with score B [n = 53]*	*Patients with score C [n = 50]*
Mean hospital stay, bed rest [days]	0	Range (1 to 3) 2	Range (5 to 9) 7
The mean time off work, [days]	Range (7 to 14) 12	Range, (12 to 17) 14	Range, (12 to 21) 13
The mean time of wound healing	Range (3 to 8)[5] weeks	Range (9 to 18 day) 14 days	Range, (8 to 17 days) 13 days
Type of anaesthesia	Local	Local or general	Spinal or general
Wound infections (%)	3 (6.3%)	3 (5.6%)	2 (4%)
Haematoma (%)	No	2 (3.7%)	3 (6%)
Recurrences			
Early [3-months] (%)	0	1 (1.9%)	1 (2%)
Late [after 3 months] (%)	1 (2.1%)	2 (3.7%)	1 (2%)
Numbness over the operation site (%)	No	5 (9.1%)	10 (20%)
Partial ischemia or flap necrosis (%)	No	No	2 (4%)

### Excision and primary closure [n = 53]

Excision and primary closure was associated with a short healing time and time off work. However, the hospital stay ranged from one to two days. Five patients (9.5%) complained of numbness over the operation site. No incidence of ischemia or marginal necrosis was observed; but we had one case with early recurrence (within three months). This case was treated conservatively without any surgical intervention. Two cases showed a late recurrence after three months of follow-up [Recurrence rate 5.6%].

### Excision with reconstructive procedures [n = 50]

We advised this procedure for patients with high scoring number from [14 to 16]. In these patients we preferred the local rotation flap, either unilateral or bilateral, for closure of the wound after the elliptical eccentric excision of all the diseased tissue. All these techniques required spinal or general anaesthesia and a week or more of bed rest in a hospital [range 5-9 days]. Ten patients (20%) complained of numbness and uneasiness over the site of surgery. There was no incidence of ischemia or flap necrosis except in two patients, in whom the necrotic area was excised [Table 2]. In one of them the wound healed by secondary intention and the other patient required a second flap after 8 weeks.

## DISCUSSION

When it comes to the management of the commonly prevalent acute presentation of the pilonidal abscess with or without a tuft of hair there is no controversy - a cruciate incision and drainage with curette to remove the necrotic tissues is all that is required and this wound is left to heal by secondary intention. Bacteriological culture, proper antibiotics and daily dressing with ever reducing packing after this simple incision and drainage of the acute sinus disease is all that is required in at least 50% of the patients.[5] Some surgeons like Ommer in 2004[6] prefer to close the abscess primary after the drainage and curettage.

The second common type of presentation is the chronic pilonidal sinus. The management of this type is controversial and opinions vary about the ideal technique of their management. Broadly there are three types of management: (1) Excision and healing by secondary intention, (2) Excision and primary closure and (3) Excision with flap cover. Regarding excision and healing by secondary intention, we preferred it for patients with Score A - this type also had no hospital stay postoperatively. The principal advantage of this technique is a low recurrence rate but this technique undoubtedly has high direct and indirect costs and the downside is a lengthy healing time (three to eight weeks).

Despite this, there is a role for wide excision in those with extensive chronic disease and those operated for failed primary closure.[7]

A modification of the standard excision is ‘marsupialisation’. The skin edges are not excised, but are sutured to the sides of the wound. This was a compromise between a completely closed and a completely opened wound and permits a some what smaller opening than does the open technique.[8] The mean healing time in a study done by Solla on 150 patients who underwent this procedure was shown to be four weeks with a recurrence rate of 6%.[9] The recurrence rate in our study was 5.6%; the improvement in our results may be advocated to the proper choice of the patients to be managed by this technique.

### Excision and primary closure

Closure of the wound is more cosmetically acceptable for some patients and is associated with a shorter healing time and time off work. However, this potential benefit is offset by the need for bed rest for up to one week in hospital coupled with a higher risk of postoperative infection. When infection intervenes the wound must be laid open and healing time is therefore longer than if the wound had been treated by secondary intention in the first place.[10] The scar can be sited over the midline or displaced laterally with one year recurrence rates of 18% and 10% respectively.[11] In another prospective study, failure of primary healing was significantly associated with early recurrence of disease.[12] In the same study the use of preoperative antibiotics did not influence the recurrence rate. Karidakis[13] pioneered a procedure raising a flap to overlap the midline with the scar sited to one side to reduce postoperative hair entry.

Bascom[15] had proposed a method to incise, drain and curette a chronic abscess through a lateral incision combined with excision of any midline pits with a minimal amount of surrounding tissue. A section of the wall of the abscess cavity opposite the incision is raised as a flap and used to close the communication between the midline pits and the abscess cavity. This is accomplished by suturing the flap to the underside of the skin bridge formed between the incision and the midline. In another study of 218 patients treated with Bascom's procedure as day cases, 6% developed a postoperative abscess requiring drainage and 10% had recurrence requiring further surgery at mean follow-up of 12.1 months (range 1-60 months).[14,15]

The local advancement flap techniques propounded by both Bascom and Karidaks were best for patients with extensive pilonidal disease, but confined to a limited area so as to avoid any closure under tension. This however required the services of senior and well trained surgeons. The recurrence rate after one year of follow-up was 5.6% versus the high incidence of recurrence of 18% that was published by Alden *et al.*[16] We attributed the improvement in the recurrence rate to the ideal choice of the operative intervention for the proper subset of patients.

### Excision with reconstructive procedures

These procedures are more technically demanding and are probably best performed by a plastic surgeon. Their use is generally restricted to recurrent complex pilonidal disease. The aim for the majority of procedures is to reshape and flatten the natal cleft to reduce friction, local warmth, moisture and hair accumulation. Many techniques have been propounded to close the defect following sinus excision – a skin flap or a flap of both skin and muscle or a skin transposition by Z-plasty or N-shaped.[17] All these techniques require general or spinal anaesthesia and a week or more of bed rest in hospital. We preferred the rotation type of local flap for coverage of these defects[18] in this sub group of patients. This type of flap had low recurrence rate; we had one case of late recurrence after eight months.

Regarding the aetiology of the sinuses Karydakis[13] had described the role of hair in the formation of pilonidal disease and divided it into three phases: Phase 1, the invader, a free hair is available to invade into a portal of entry into the skin; Phase 2, the force that causes the insertion; and Phase 3, the vulnerability of the skin to the insertion of hair at the depth of the natal cleft. This theory means that there is presence of the hair in all sinuses but this disagrees with our finding that only 40% of the sinuses contained hair at surgery. However, in another observation on specimens following wide excision Lineaweaver *et al.*[14] has shown that the pits penetrating into the dermis were distended with keratin and debris, but not all had hairs. Hair entry into the follicles by a variety of means was originally thought to be the primary event in the development of pilonidal sinus disease. However, there was evidence to suggest that the enlargement of the follicles precedes hair gaining access and at operation hair was found in only half of the cases.[15] This is in agreement with our results regarding the presence of the sinuses without hair accumulation.

We believe that the cause of pilonidal sinuses can be either congenital or acquired; and it is initiated by congenital pilonidal dimples or pits that we observed were present in some individuals at the top of the crease between the buttocks, about 7-9cm from the anal orifice. Those patients with the dimples were considered patients with potential pilonidal sinus disease and after adolescence the pits or dimples enlarged and became wide enough to create a portal of entry for cellular debris or free hairs. Loose hairs are drilled, propelled, and sucked into these pilonidal pits by friction and movement of the buttocks whenever a patient stands or sits. Hair enters tip first, and the barbs on the hair prevent it from being expelled so that the hair becomes entrapped. This trapped hair stimulates a foreign body reaction and infection. The precipitating factors of this pathology are obesity and the process of repeatedly sitting and standing may cause the hairs to be attracted or sucked into the site of the pilonidal dimple or pit leading to cyst formation. There are many factors that support this theory like the high recurrence rate of the disease in spite of wide local excision and flap coverage if associated with improper excision of the pilonidal dimple. One has to remember that not all hairy individuals had pilonidal sinus disease. Furthermore, the presence of the sinus in non hairy or slightly hairy individuals like females is also not very clearly understood.

Finally, in our opinion, three factors should exist to intiate the pathology of the pilonidal sinus disease – the first is the presence of a well-stimulated [after adolescence] pilonidal dimple as a congenital cause; the second factor is accumulation of free hairs or cellular debris in the dimple; and the third factor is the power to introduce and charge the area to initiate the pathology. Thus we believe that midline scar alone without a nidus for initiating the pathology [dimple or pit] is of no significant value as a precursor for recurrence – the more important factors being the presence of sutures, depressions or dimples in the midline scar.

Recurrence can be divided into two groups: Early and Late. Early recurrence is usually due to failure to identify one or more sinuses during surgery. Late recurrence is usually due to secondary infection caused by residual hair or debris in the pilonidal dimple that was not removed at operation, as well as inadequate wound care or insufficient attention to depilation.[19]

## CONCLUSION

This simple scoring system allowed an easy and rapid decision making for management of pilonidal sinus diseases with satisfactory results.
